# Supplementary material for: End Sequence Analysis Toolkit (ESAT) expands the extractable information from single-cell RNA-seq data
Source: Genome Res. 2016 Oct;26(10):1397–410. doi: 10.1101/gr.207902.116 (PMC5052061; doi:10.1101/gr.207902.116)
Supplement: Supplemental Material [file supp_gr.207902.116_Supp_Materials.tar.gz › ESAT-esat_v0.1/doc/html/functions_r.html]

ESAT: Class Members


ESAT


- Main Page
- Related Pages
- Packages
- Classes
- Files

- Class List
- Class Index
- Class Hierarchy
- Class Members

- All
- Functions
- Variables
- Enumerator

- a
- b
- c
- d
- e
- f
- g
- h
- i
- j
- k
- l
- m
- n
- o
- p
- q
- r
- s
- t
- u
- v
- w
- z


All Classes Namespaces Files Functions Variables Enumerator Pages

Here is a list of all class members with links to the classes they belong to:

### - r -

- random
  : broad.pda.seq.protection.MultiSampleScanPeakCaller
  , Jama.Matrix
- RANDOM\_SEED
  : broad.pda.seq.rap.BuildRatioNullDistribution
  , broad.pda.seq.rap.ShuffleBAM
- randomPermutation()
  : broad.core.math.Statistics
- randomPermuteColumns()
  : broad.core.datastructures.MatrixWithHeaders
- rank()
  : broad.core.datastructures.MatrixWithHeaders
  , broad.core.math.MannWhitney
  , broad.core.math.Statistics
  , Jama.Matrix
  , Jama.SingularValueDecomposition
- rankOrder()
  : broad.core.math.MannWhitney
- RankOrder()
  : broad.pda.geneexpression.clustering.RankOrder
- RAPPeakCaller()
  : broad.pda.seq.rap.RAPPeakCaller
- RatioScore()
  : umms.core.model.score.RatioScore
- rawRegions()
  : broad.pda.seq.segmentation.ContinuousDataAlignmentModel
- read()
  : Jama.Matrix
  , umms.core.annotation.AnnotationFileReader
  , umms.core.general.TabbedReader
- READ\_LIST\_FILE
  : broad.pda.samtools.FilterFastqReads
- readCigarFlag
  : umms.core.writers.PairedEndWriter
- readDstFile()
  : broad.core.math.Loess
- ReadEndsInFilter()
  : umms.core.readFilters.ReadEndsInFilter
- ReadSimulator2()
  : broad.pda.seq.protection.ReadSimulator2
- readSiphyOutToBed()
  : broad.pda.annotation.BEDFileParser
- readStartFlag
  : umms.core.writers.PairedEndWriter
- ReadStartsInFilter()
  : umms.core.readFilters.ReadStartsInFilter
- reconstructGeneEnds()
  : umms.esat.ESAT
  , umms.esat.ESATDistanceCalculator
- ReconstructionComparer()
  : broad.pda.seq.segmentation.utils.ReconstructionComparer
- reduceToDifference()
  : broad.core.annotation.BasicGenomicAnnotation
- REF
  : broad.pda.seq.rap.DistanceToBED
- REFERENCE\_SEQUENCE
  : broad.pda.seq.rap.GCContent
- refreshAttributeMap()
  : umms.core.alignment.AbstractPairedEndAlignment
- refreshPvalue()
  : umms.core.model.score.BinomialEnrichmentScore
  , umms.core.model.score.CountScore
- refreshScanPvalue()
  : umms.core.model.score.ScanStatisticScore
- REG\_PATTERN
  : broad.core.annotation.BasicGenomicAnnotationReader
- REGION
  : broad.pda.seq.rap.GenomeCommandLineProgram
- region
  : umms.core.coordinatesystem.ShuffledIterator< T extends Annotation >
- registerFormat()
  : jaligner.formats.FormatFactory
- remove()
  : broad.core.datastructures.IntervalTree< V >.FwdIterator
  , broad.core.datastructures.IntervalTree< V >.OverlapIterator
  , broad.core.datastructures.IntervalTree< V >
  , broad.core.datastructures.IntervalTree< V >.RevIterator
  , broad.core.datastructures.JCSCache.JCSNodeIterator
  , broad.core.datastructures.ReversibleIterator< E >
  , broad.core.multiplealignment.MultipleAlignment
  , broad.core.sequence.WindowSlider
  , broad.pda.annotation.BEDFileParser
  , broad.pda.samtools.PairedEndReadIterator
  , broad.pda.samtools.WrappedIterator
  , broad.pda.seq.fastq.FastqParser
  , broad.pda.seq.segmentation.WrappedIGVIterator
  , umms.core.coordinatesystem.ShuffledIterator< T extends Annotation >
  , umms.core.general.CloseableFilterIterator< T >
  , umms.core.model.AlignmentModel.FilteredIterator
  , umms.core.model.AlignmentModel.NodeIterator
  , umms.core.model.AlignmentModel.WrapAlignmentCountIterator
  , umms.core.model.JCSAlignmentModel.FilteredIterator
  , umms.core.model.JCSAlignmentModel.NodeIterator
  , umms.core.model.JCSAlignmentModel.WrapAlignmentCountIterator
  , umms.core.model.score.WindowScoreIterator< T extends WindowScore >
- REMOVE\_MATE
  : broad.pda.samtools.FilterPairedReadsByMAQ
- removeAnnotation()
  : umms.core.annotation.AnnotationList< T extends Annotation >
- removeAtEnd
  : broad.pda.samtools.TrimFastqReads
- removeAtStart
  : broad.pda.samtools.TrimFastqReads
- removeAtSymbolFromName()
  : broad.pda.seq.fastq.FastqSequence
- removeEdge()
  : broad.pda.seq.graph.ChromosomeWithBubblesJGraphT
  , broad.pda.seq.graph.Path
- removeExtraScores()
  : broad.core.annotation.BasicLightweightAnnotation
  , broad.core.annotation.LightweightGenomicAnnotation
  , broad.core.sequence.SequenceRegion
- removeFilter()
  : umms.core.model.AlignmentModel
  , umms.core.model.JCSAlignmentModel
- removeInterval()
  : umms.core.annotation.CompoundInterval
- removeOverlappingRows()
  : broad.pda.datastructures.LocationAwareMatrix
- removeSingeleExon()
  : broad.pda.annotation.BEDFileParser
- removeSmallTranscripts()
  : broad.pda.annotation.BEDFileParser
- removeSpanningGapNodes()
  : broad.pda.seq.graph.ChromosomeWithBubblesJGraphT
- removeVertex()
  : broad.pda.seq.graph.ChromosomeWithBubblesJGraphT
- RepeatMaskerAnnotation()
  : broad.core.annotation.RepeatMaskerAnnotation
- repeatMaskerAnnotationFactory
  : broad.core.annotation.AnnotationFactoryFactory
- RepeatMaskerAnnotationReader()
  : broad.core.annotation.RepeatMaskerAnnotationReader
- replaceFileExtension()
  : broad.core.util.CLUtil
- reset()
  : broad.core.math.CombinationGenerator
- resetPI()
  : broad.core.siphy.EvolutionaryModel
- resetProbWithNullPriorProb()
  : broad.core.motif.PositionWeightColumn
- resetToUniformProb()
  : broad.core.motif.PositionWeightColumn
- resetTreeCache()
  : broad.pda.seq.segmentation.AlignmentDataModel
  , broad.pda.seq.segmentation.AlignmentDataModelStats
  , broad.pda.seq.segmentation.ContinuousDataAlignmentModel
  , broad.pda.seq.segmentation.GenericAlignmentDataModel
- restartIterator()
  : broad.pda.seq.segmentation.AlignmentDataModel
  , broad.pda.seq.segmentation.GenericAlignmentDataModel
- RestrictionEnzyme()
  : broad.core.primer3.RestrictionEnzyme
- reverse()
  : broad.core.multiplealignment.MAFAlignment
  , broad.core.multiplealignment.MultipleAlignment
  , broad.core.primer3.ComputeMIRScore
  , broad.core.sequence.Sequence
- reverseComplement()
  : broad.core.motif.PositionWeightMatrix
  , broad.core.primer3.ComputeMIRScore
  , broad.pda.rnai.ExtractSequence
- reverseIterator()
  : broad.core.datastructures.IntervalTree< V >
- reverseSequence()
  : broad.core.sequence.Sequence
- ReversibleIterator()
  : broad.core.datastructures.ReversibleIterator< E >
- RevIterator()
  : broad.core.datastructures.IntervalTree< V >.RevIterator
- RIGHT\_MARGIN
  : broad.core.annotation.BasicGenomicAnnotationReader
- rightIntersect()
  : broad.core.math.EmpiricalDistribution
- RNAiGeneAnnotation()
  : broad.pda.rnai.RNAiGeneAnnotation
- roll()
  : broad.core.math.UnfairDie
- roots()
  : broad.core.math.MathUtil
- round()
  : broad.core.datastructures.MatrixWithHeaders
- rowDensityNormalization()
  : broad.core.datastructures.MatrixWithHeaders
- rowDimension()
  : broad.core.datastructures.MatrixWithHeaders
- RPIDecomposition()
  : broad.core.siphy.EvolutionaryModel
- RPKM\_OFFSET
  : umms.core.model.score.NewRatioScore
  , umms.core.model.score.RatioScore
  , umms.core.model.score.WindowAllScore
- RPKM\_RATIO\_OFFSET
  : broad.pda.seq.rap.SlideAndCalculate
- runBackwardAlgorithm()
  : broad.core.hmm.MarkovModel< T >
  , broad.core.siphy.PiHMM
- RunDifferentialExpression()
  : broad.pda.differentialExpression.RunDifferentialExpression
- runForwardAlgorithm()
  : broad.core.hmm.MarkovModel< T >
  , broad.core.siphy.PiHMM
- runForwardAlgorithmNoRescaling()
  : broad.core.hmm.MarkovModel< T >
- RunGSEA()
  : broad.pda.differentialExpression.RunGSEA
- runKmeans()
  : broad.pda.geneexpression.clustering.Kmeans
- RuntimeIOException()
  : umms.core.exception.RuntimeIOException
- RunTimeSeries()
  : broad.pda.differentialExpression.RunTimeSeries


---

Generated on Fri Aug 15 2014 15:11:16 for ESAT by  

 1.8.7
